# Effect of optimized germination on nutritional functional and phytochemical characteristics of green gram

**DOI:** 10.1038/s41598-026-42908-y

**Published:** 2026-03-17

**Authors:** Thirukkumar Subramani, Sivasankari Ruckmangathan, Hemalatha Ganapathyswamy, Sivasubramanin Palanisamy, Murugesan Palaniappan, Subramanian Lakshmi Sankar, Mohamed Abbas, Shaeen Kalathil, Mezigebu Belay

**Affiliations:** 1 Department of Food Science, Amrita School of Agricultural Sciences, Amrita Vishwa Vidyapeetham, Coimbatore, 642109 Tamil Nadu India; 2 Department of Food Science and Nutrition, Community Science College and Research Institute, Tamil Nadu Agricultural University, Madurai, 625104 Tamil Nadu India; 3 Department of Mechanical Engineering, School of Engineering, Mohan Babu University, Tirupati, 517102 Andhra Pradesh India; 4 Department of Mechanical Engineering, College of Engineering, Imam Mohammad Ibn Saud Islamic University (IMSIU), Riyadh, 11432 Kingdom of Saudi Arabia; 5 Department of Mechanical Engineering, Sathyabama Institute of Science and Technology, Chennai, Tamil Nadu India; 6 Department of Electrical Engineering, College of Engineering, King Khalid University, 61421 Abha, Saudi Arabia; 7 Department of Condensed Matter Physics, Saveetha School of Engineering, Saveetha Institute of Medical and Technical Sciences (SIMATS), Thandalam, Chennai, Tamil Nadu India; 8 Department of Electrical Engineering, College of Engineering, Princess Nourah bint Abdulrahman University, P.O. Box 84428, 11671 Riyadh, Saudi Arabia; 9 Department of Metallurgical and Materials Engineering, College of Engineering, Ethiopian Defence University, 1041 Bishoftu, Ethiopia; 10 Department of Food Processing Technology and Management , PSGR Krishnammal College for Women, Coimbatore, Tamil Nadu, India

**Keywords:** Green gram, Optimized germination, Sensory optimization, Phytochemicals, Functional properties, Anti-nutritional factors, Vitamin C, Functional foods, Biochemistry, Plant sciences

## Abstract

Germination is an effective bioprocessing strategy for enhancing the nutritional quality and functional potential of legumes. This study systematically evaluated the effects of controlled germination on the nutritional, functional, phytochemical, antioxidant, and sensory characteristics of ten elite green gram (*Vigna radiata* L.) cultures, with the objective of identifying genotypes suitable for sprout-based functional food applications. Germination was conducted for 8, 12, 16, and 20 h, and optimization was achieved based on overall sensory acceptability (OSA) score using a nine-point hedonic scale. The highest sensory acceptability scores, ranging from 6.01 to 8.60, were observed at 8 - 12 h of germination, whereas extended germination significantly reduced acceptability due to the development of bitterness. Compared with non-germinated samples, optimally germinated green gram cultures exhibited significant improvements in nutritional composition, including increased crude protein content from 17.38 – 24.81 to 20.12 - 26.32 g 100 g⁻^1^ and crude fiber from 9.86 - 13.82 to 11.36 - 16.42 g 100 g⁻^1^. Ash content also increased marginally following germination. Vitamin C, which was absent in raw grains, was synthesized during germination and reached levels of approximately 55.04 to 85.48 mg 100 g⁻^1^. In contrast, anti-nutritional factors were substantially reduced, with tannin content decreasing from 320 - 458 to 65 - 97 mg tannic acid equivalent 100 g⁻^1^ and phytic acid from about 754 - 906 to 102 - 175 mg 100 g⁻^1^. Germination significantly enhanced phytochemical composition and antioxidant capacity, as evidenced by increased total phenolic and flavonoid contents and higher DPPH radical scavenging activity, which increased from 23.78 - 32.41% in raw grains to 35.47 - 40.59% in germinated samples. Functional properties, including water and oil absorption capacities, were also significantly improved following germination. Overall, this study presents a novel comparative screening of elite green gram cultures and establishes optimized germination as a practical and scalable approach for developing green gram sprouts in both fresh and dry forms in salads, weaning/supplementary foods, nutrient-dense convenience foods, etc., for improved nutrition.

## Introduction

Pulses serve as a primary protein for vegetarians across the globe and provides significant amounts of B vitamins, minerals, omega-3 fatty acids and dietary fiber. Compared with animal foods, which have a high level of saturated fat, pulses are healthy sources of protein, predisposing individuals to an increased risk of colon cancer and cardiovascular diseases^1,2^.

The consumption of pulses by processing is a traditional practice that includes soaking, germination, fermentation, and cooking. Germination by different pulses results in pulses of high nutritional value tshrough the elimination/reduction of anti-nutrition compounds^1^. Ascorbic acid, a fresh food vitamin, is solely present in fresh fruits and vegetables. Among food grains, particularly pulses, germination or sprouting initiates ascorbic acid synthesis, decreases anti-nutrients, and enhances protein bioavailability and nutrient utilization, which leads to disease prevention and promotes health benefits. The germination of green grams significantly increases antioxidant activity through the production of ascorbic acid^3,4^. Additionally, the synthesis of ascorbic acid using reducing sugars during the sprouting process affects sensory characteristics^5^.

Germination triggers enzymatic activities that improve the bioavailability of minerals like iron, zinc, and calcium, while lowering the concentration of phytate and tannin^6–8^. Additionally, germination also increases the levels of bioactive compounds like gamma-aminobutyric acid (GABA), flavonoids and phenolic acids, which contribute to improved antioxidant capacity and functional food potential^9–11^. Furthermore, flavour active compounds, such as pyrazines and phenolic acids, play a crucial role in determining taste and aroma in plant-based foods, and their concentrations can be modified through various processing methods such as germination, soaking and fermentation. Multi omics approaches revealed that pyrazines, aldehydes and sulfur compounds formed during germination significantly contribute to the sensory characteristics of legumes and their concentrations can be optimized to reduce undesirable beany or bitter taste^12,13^.

In addition, a suitable variety of pulse cultivation methods is very important in the agricultural sector and thus promotes the dual benefits of farmers and consumers. Identifying and selecting cultivars with lower levels of undesirable flavour compounds has been proposed to enhance both the nutritional quality and sensory attributes of the pulses. Genetic studies have identified key loci associated with flavor intensity, bitterness, and seed coat perception in legumes, suggesting that breeding for improved sensory traits is feasible and can be guided by molecular markers^14^.

Globally, sprouted green grams (*Vigna radiata* L.) is gaining attention due to its low anti-nutritional contents, improved digestibility and rich vitamin C content. Understanding the effectiveness of germination on nutritional parameters will contribute valuable insights for promoting health benefits, disease prevention, and the formulation of novel food products tailored for infants and elderly. Furthermore, the identification of suitable green gram cultures for sprouting may play a significant role in improving the nutritional and sensory quality of plant-based diets. In this context, screening elite green gram cultures for their suitability as nutrient-enriched consumable forms of green gram sprouts was considered of interest, as was investigating the effectiveness of germination on phenols, flavonoids and the total antioxidant activity of raw and sprouted samples of selected green gram cultures. Despite extensive studies on germination and its impact on the nutritional and sensory quality of pulses, comparative evaluations of multiple green gram cultivars for their potential to enhance phenolic, flavonoid, and antioxidant content, along with sensory acceptability, remain limited. This study is novel in systematically screening ten elite green gram cultures to identify those with optimal germination potential for nutrient enrichment and improved sensory attributes, thereby providing insights for both functional food development and cultivar selection for targeted health benefits.

## Materials and methods

### Material

Ten promising green gram (*Vigna radiata*) cultures, namely, VGG 17-109 (GG21), COGG 17-002 (GG22), VGG 16-047 (GG23), COGG 980 (GG24), VGG 17-076 (GG25), CO 7 (GG26), NBW 4 (GG27), VGG 16-058 (GG28), CO 8 (GG29) and VGG 18-003 (GG30), were selected for the study. The selected green gram cultures were high yielding, short in duration had synchronous maturity and were suitable for rain-fed cultivation because they required less water for cultivation. The chosen green gram cultures were obtained from the National Pulses Research Center, Tamil Nadu Agricultural University, Vamban, Tamil Nadu, India. The cultures were screened for foreign substances and damaged seeds and stored in refrigerated conditions. The chemicals used for this study were purchased from Sigma - Aldrich (Bangalore, India) and Hi Media Laboratories Pvt. Ltd. (Mumbai, India).

### Germination process

The selected green gram cultures were washed with deionized water for 5 min. The pre-soaked seeds were drained and subsequently immersed in deionized water at 32±2°C for a duration of 4 hours, followed by germination at 38±2°C for 8, 12, 16 and 20 hours in an incubator (M/s. Lark instruments, Chennai) as described by Liu *et al.*^15^

### Physical characteristics

Three samples from each green gram culture were randomly selected and assessed for physical characteristics, *viz*., 100-grain weight, length, and breadth, before and after the germination process^3^. The total sprout length of germinated green gram was measured by combining the sprouted radicle length and seed length. Both the length and breadth of the samples were measured using a digital Vernier caliper (M/s. Zhart Vernier Caliper Digital) with an accuracy of 0.01 mm. The mean value of ten determinations was reported.

## Nutrient characteristics

Moisture, crude protein, crude fat, crude fiber, and ash levels of the raw and germinated green gram cultures were assessed using AACC methods 44-01.01, 46-13.01, 30.20.01, 32-10.01 and 08.01.01, respectively^16^. Total carbohydrate concentrations were estimated by the anthrone method^17^. The vitamin C concentration was estimated as per the method of AOAC 967.21^18^.

### Anti-nutritional characteristics

The tannin content was estimated as per method^17^ and expressed as mg of tannic acid equivalent (TAE) 100 g^-1^ of sample. The phytate content was analyzed by method^19^ and the sample readings were calibrated with the standard curve of phytic acid.

### Functional characteristics

The raw green gram cultures were ground to flour using a mini flour mill (M/s Amstrad, India), and the germinated green gram cultures were dried (60°C for 8 h), pulverized to flour and sieved through a 60 BS metric sieve. The functional characteristics of the flours from the raw and germinated green cultures were analyzed, namely, water absorption capacity (WAC), oil absorption capacity (OAC)^20^, emulsifying activity (EA)^21^, gelation capacity (GC)^22^ and foaming capacity^23^.

### Phytochemical characteristics

The total phenolic content was determined using the Folin–Ciocalteu reagent method^24^ and expressed as gallic acid equivalents (GAE) (mg 100 g^-1)^ and ferulic acid equivalents (FAE) (mg 100 g^-1)^. Total flavonoids were quantified by the aluminum chloride colorimetric assay^25^, with results presented as quercetin acid equivalents (QAEs) (mg 100 g^-1^) and catechin acid equivalents (CAEs) (mg 100 g^-1^). Antioxidant activity was assessed by the 2,2,diphenyl-1-picrylhydrazyl (DPPH) assay^26^^,^ and the values were reported as the percentage of radical scavenging activity (% RSA) of the methanolic extracts.

### Sensory characteristics

Green gram cultures were germinated for different durations (8, 12, 16 and 20 h) and evaluated for sensory attributes such as colour, appearance, flavor, texture, taste, and overall acceptability^27^. The sensory assessment was performed by 20 semi-trained panelists using a nine-point hedonic scale (1 = dislike extremely to 9 = like extremely). The overall sensory acceptability (OSA) score obtained from this evaluation was used to determine the optimized sample.

### Statistical analysis

All experiments were carried out in triplicate, and the outcomes are expressed as mean values ± standard deviation (SD). Statistical evaluation was conducted through one-way analysis of variance (ANOVA), and mean differences among treatments were identified using Duncan’s multiple range test and post-hoc Tukey analysis at a significance level of *p* < 0.05. Data analysis was performed using SPSS software, version 21.0 (IBM SPSS Inc., Chicago, IL).

## Results and discussion

### Optimization of the germination time of green gram cultures by sensory analysis

The effect of germination time on OSA is presented in Table 1. The OSA score ranged between 6.01 and 8.60 out of 9.0, which indicated that the sprouted green gram samples had acceptability scores ranging from “like extremely” to “like slightly” corresponding to different periods of germination. The GG21, GG22, GG23, GG24, GG29 and GG30 cultures had high OSA scores (7.40 to 8.60/9.0) after 8 hours of germination. Alternatively, GG25, GG26, GG27 and GG28 had OSA scores in the range of 7.14 to 8.32/9.0 after 12 h of germination. Post-hoc Tukey analysis revealed that overall sensory acceptability was highest at 8 - 12 h of germination and declined significantly at 16 h and 20 h across all green gram cultures (*p* < 0.05). As germination time increased, the OSA score dropped because the sprouts developed an altered appearance and a mild bitterness. The improved overall sensory acceptability observed in optimally germinated green gram can be attributed to germination-induced biochemical modifications, including enzymatic hydrolysis of macromolecules, reduction of beany and bitter flavour precursors, and formation of desirable flavour-active compounds. Similar mechanisms have been reported for germinated black gram^2^ and cowpea^28^, where controlled sprouting enhanced palatability, texture, and aroma through the breakdown of anti-nutritional factors and modulation of volatile and phenolic profiles, resulting in higher consumer acceptance. The highest OSA score obtained with a suitable germination time of 8 h in the green gram cultures was selected for further study. The moisture content, vitamin C content, total antioxidant activity, total phenol content, total flavonoids, 100-grain weight and sprout length were analyzed in the fresh green gram sprout samples, and other parameters, such as total carbohydrates, crude protein, crude fiber, crude fat, total ash and functional characteristics, such as WAC, OAC, GC, EA, and GC, were analyzed in the dried samples.

**Table 1:** Effect of germination time on the overall sensory acceptability score of selected green gram cultures

**Green gram culture**	**Germination time (h)**			
	**8**	**12**	**16**	**20**
GG21	7.56±0.15^bx^	7.25±0.04^dy^	7.10±0.15^cy^	6.48±0.51^cz^
GG22	7.40±0.07^cx^	7.21±0.13^dy^	7.14±0.35^cy^	6.37±0.68^cz^
GG23	7.70±0.06^bx^	7.15±0.28^dy^	7.00±0.54^cy^	6.34±0.14^cz^
GG24	8.14±0.26^ax^	8.04±0.46^ax^	7.58±0.25^by^	7.04±0.21^az^
GG25	7.46±0.21^bx^	7.68±0.13^cx^	7.14±0.37^cy^	6.12±0.17^dz^
GG26	7.57±0.16^bx^	7.96±0.18^bx^	7.21±0.64^cy^	6.06±0.51^ez^
GG27	6.75±0.08^dx^	7.14±0.15^dx^	6.48±0.52^dy^	6.01±0.36^ez^
GG28	7.98±0.51^abx^	8.32±0.02^ax^	7.56±0.45^ay^	7.14±0.26^az^
GG29	8.32±0.25^ax^	8.01±0.53^abx^	7.52±0.38^ay^	7.10±0.34^az^
GG30	8.60±0.29^ax^	8.13±0.41^ax^	7.64±0.52^ay^	6.95±0.08^bz^

Values are expressed as mean ± SD (n = 3). Different superscript letters within the same column (a - e) indicate significant differences among green gram cultures, while different superscript letters within the same row (x - z) indicate significant differences among germination times, as determined by one-way ANOVA followed by Tukey’s HSD test at *p* < 0.05.

### Physical characteristics of the selected gram-green cultures

**Fig 1:**
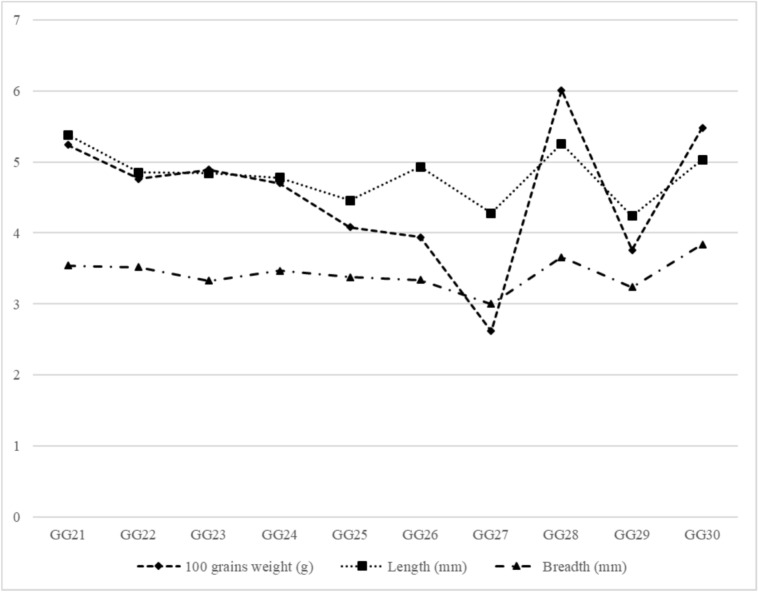
Physical characteristics of non-germinated green gram cultures; Each value is the mean of three replicate analyses (n=3)

**Fig 2:**
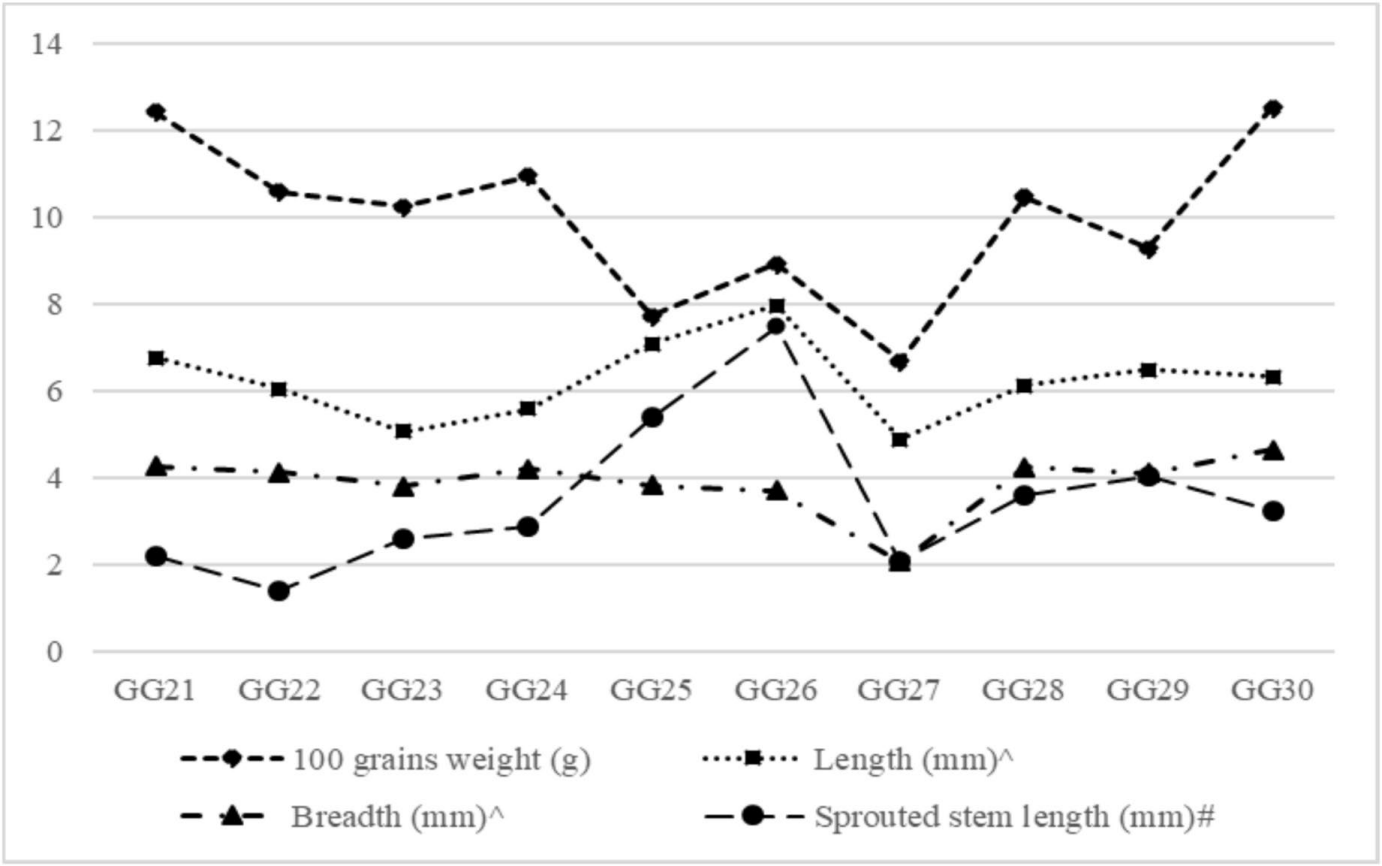
Physical characteristics of germinated green gram cultures; # Fresh sprouted green gram samples; ^including sprouted stem length and breadth in sprouted green gram; Each value is the mean of three replicate analyses (n=3)

The physical characteristics of the raw and optimized sprouted green gram cultures are shown in Figs. 1 and 2, respectively. The 100-grain weight was high in the GG30 green gram culture (5.48 g), which was significantly different (*p<0.05*) from that in the other cultures, and lowest in the GG27 culture (2.62 g). The length and breadth of the selected green gram cultures ranged from 4.28 to 5.38 mm and 3.00 to 3.66 mm, respectively. The maximum and minimum widths were obtained for the GG30 (3.84 mm) and GG26 (3.00 mm) cultures, respectively.

The 100-grain weight, length and breadth of the sprouted green gram cultures ranged from 7.72 to 12.53%, 4.88 to 7.96 mm, and 3.71 to 4.64 mm, respectively and a significant difference (*p<0.05*) was observed between the raw and sprouted green gram cultures. These values suggest that the initial grain size and boldness of raw green gram significantly influence the dimensional characteristics of sprouted grains. Larger and bolder grains tend to exhibit greater increases in length and breadth during sprouting due to enhanced water absorption, seed coat loosening, and active cell elongation associated with germination. Murugkar et al.,^29^ noted that seed size affects hydration behavior and subsequent morphological changes during sprouting in soybean, have reported similar observations. Likewise, Devi et al.^30^ demonstrated that larger cowpea seeds showed more pronounced dimensional expansion during sprouting, attributed to metabolic activation and reserve mobilization, supporting the present findings in green gram. Changes in sprouting characteristics in all cultures may occur through the aging of the embryo or changes in the remainder of the seed^31^. The variation in terms of 100-grain weight, length, and breadth of the grains observed among the green gram cultures was generally influenced by varietal differences, cultivation practices, and moisture content of the grain during the postharvest period. The average length, width, and thickness of the green grains were 4.21, 3.17, and 3.08 mm, respectively^32^, which could explain why the increase in the grain moisture content during the germination process influenced the length, width, and thickness of the grains.

### Nutrient characteristics of selected green gram cultures

The nutrient characteristics of the raw (non-germinated) and optimized germinated samples of the selected green gram cultures are presented in Table 2 and Table 3, respectively. In the germinated samples, except for moisture content and vitamin C content, the other nutrients, anti-nutrients, and phytochemicals were equalized with the respective raw green gram samples. The moisture content of the raw green gram cultures ranged between 12.58 and 15.24%. Among the optimized germinated green gram samples, the lowest moisture content was 33.47%, which was observed in GG25 and was on par with that in GG26, GG27 and GG29. The highest moisture content was observed in sample GG24 (44.73%). The moisture content of the sprouted green gram cultures increased in the range of 122.39% to 234.06% compared with that of the non-germinated green gram samples.

**Table 2:** Nutrient and anti-nutritional characteristics of non-germinated green gram cultures

**Green gram culture**	**Nutrient content (100 g**^**-1**^**)**						**Anti-nutrient content (100 g**^**-1**^**)**	
	**Moisture content (%)**	**Carbohydrate** **(g)**	**Crude Protein(g)**	**Crude Fat** **(g)**	**Crude Fiber (g)**	**Ash** **(g)**	**TAE (mg)**	**Phytic acid (mg)**
GG21	15.24±0.57^c^	59.58±0.86^b^	20.52±0.00^f^	2.09±0.06^d^	12.46±0.08^b^	2.31±0.01^b^	354±10.12^d^	902±24.55^a^
GG22	14.85±0.42^c^	52.53±0.71^c^	23.48±0.38^b^	2.29±0.04^e^	13.48±0.19^a^	2.04±0.06^cde^	320±7.16^e^	817±20.70^c^
GG23	14.67±0.01^c^	60.32±0.13^a^	17.38±0.18^g^	2.16±0.03^d^	12.65±0.30^b^	2.08±0.00^cd^	332±0.67^e^	852±27.24^b^
GG24	13.39±0.53^b^	59.85±1.56^ab^	21.54±0.83^e^	1.86±0.07^ab^	12.53±0.28^b^	1.98±0.02^e^	368±1.00^d^	786±14.97^d^
GG25	15.05±0.65^c^	55.91±1.81^c^	22.04±0.37^de^	2.49±0.01^f^	12.87±0.42^b^	2.13±0.08^c^	400±8.70^bc^	865±4.70^b^
GG26	13.80±0.34^b^	54.29±1.43^c^	21.86±0.64^e^	2.79±0.03^g^	10.53±0.30^d^	2.23±0.08^b^	458±10.95^a^	883±1.80^a^
GG27	14.63±0.01^c^	51.44±1.75^c^	24.81±0.17^a^	2.15±0.03^d^	11.74±0.16^c^	1.56±0.06^g^	385±8.12^c^	754±0.51^d^
GG28	14.70±0.34^c^	53.4±1.04^c^	23.08±0.04^b^	1.79±0.03^a^	9.86±0.42^e^	2.03±0.02^de^	367±9.98^d^	813±17.14^c^
GG29	14.69±0.63^c^	54.82±0.66^c^	22.74±0.10^cd^	1.92±0.00^c^	12.44±0.35^b^	1.87±0.04^f^	422±4.30^b^	765±7.80^d^
GG30	12.58±0.12^a^	59.21±1.20^c^	19.97±0.57^f^	1.87±0.04^ab^	13.82±0.53^a^	2.42±0.04^a^	432±9.99^b^	906±15.41^a^

Each result is expressed as the mean ± standard deviation (SD) of three independent replicates (n = 3). Superscript letters indicate values within the same column that differ significantly at *p* < 0.05.

**Table 3:** Nutrient and anti-nutritional characteristics of optimized germinated green gram cultures

**Green gram culture**	**Nutrient characteristics (100g**^**-1**^**)**							**Anti-nutrient content (100g**^**-1**^**)**	
	**Moisture content**^**#**^** (%)**	**Carbohydrate (g)**	**Crude Protein (g)**	**Crude Fat (g)**	**Crude Fiber (g)**	**Ash** **(g)**	**Vitamin C**^**#**^ **(mg)**	**TAE (mg)**	**Phytic acid (mg)**
GG21	38.47±0.06^d^	50.28±0.75^f^	23.18±0.65^d^	0.96±0.01^e^	14.07±0.41^c^	2.33±0.07^b^	62.06±1.90^e^	76±0.08^d^	154±4.08^b^
GG22	38.61±1.53^d^	41.18±0.59^a^	24.58±0.15^c^	0.52±0.01^a^	13.86±0.36^c^	2.05±0.06^de^	84.28±3.49^a^	86±2.45^b^	168±5.48^a^
GG23	37.03±0.73^bc^	49.65±0.17^f^	20.12±0.12^e^	0.60±0.01^b^	13.86±0.23^c^	2.14±0.05^cd^	66.83±2.04^c^	88±0.65^b^	175±2.85^a^
GG24	44.73±0.16^f^	49.43±1.64^f^	23.34±1.00^d^	0.54±0.00^a^	14.33±0.10^c^	1.99±0.03^e^	71.40±1.67^b^	80±2.23^c^	132±2.42^c^
GG25	33.47±0.24^a^	47.03±1.99^cd^	26.31±0.78^a^	1.3±0.06^f^	16.42±0.26^a^	2.18±0.03^c^	65.41±0.41^de^	91±1.54^ab^	163±1.88^ab^
GG26	34.75±0.09^a^	48.52±0.34^cde^	25.57±0.13^ab^	1.4±0.01^g^	15.38±0.34^b^	2.31±0.03^b^	72.24±1.95^b^	88±1.25^b^	102±0.13^e^
GG27	33.99±0.21^a^	43.40±1.21^b^	26.32±0.83^a^	0.69±0.01^c^	14.04±0.35^c^	1.61±0.06^f^	85.48±2.61^a^	72±0.34^d^	114±0.38^d^
GG28	39.17±1.58^e^	46.76±1.81^c^	25.16±0.04^bc^	0.71±0.02^c^	11.36±0.49^d^	2.05±0.02^de^	56.83±1.53^f^	65±0.30^e^	122±1.41^d^
GG29	34.82±0.72^a^	46.42±1.63^c^	25.30±0.20^bc^	0.83±0.01^d^	15.64±0.62^b^	1.95±0.01^e^	55.04±1.33_f_	97±2.04^a^	146±1.39^c^
GG30	36.44±1.60^b^	49.16±0.57^de^	23.14±0.14^d^	0.61±0.01^b^	15.98±0.21^ab^	2.50±0.09^a^	66.40±1.07^c^	94±2.49^a^	159±5.30^b^

# Fresh sprouted green gram samples; TAE – Tannic Acid Equivalent; Each result is expressed as the mean ± standard deviation (SD) of three independent replicates (n = 3). Superscript letters indicate values within the same column that differ significantly at *p* < 0.05.

The total carbohydrate content ranged from 51.44 to 60.32 g 100 g^-1^ and 41.18 to 50.28 g 100 g^-1^ in the raw and optimized germinated green gram cultures, respectively. These values correspond with the carbohydrate content values of green gram samples^33^ and were lower than those of the other samples^34^. Among the sprouted green gram cultures, the carbohydrate content varied significantly (*p<0.05*) between the optimized green gram samples, which was consistent with the values of the raw green gram cultures; however, a high variation was observed in GG22 (21.61% greater than that in the non-germinated samples), and the lowest variation was observed in GG26 (10.63% greater than that in the non-germinated samples). During the early stages of germination, a large amount of soluble sugars are used during respiration, and alpha-amylase is not synthesized to hydrolyze starch, leading to a decrease in sugars^35,36^.

The crude protein concentration ranged from 17.38 to 24.81 g 100 g^-1^ in the non-germinated green gram samples. These values are in accordance with the protein values reported for different green gram cultivars^34,37^. The range of protein content for the optimized germinated green gram was 20.12 g 100 g^-1^ in GG23 to 26.32 g 100 g^-1^ in GG27. Similarly, compared with those of non-germinated green plants, the percentage of change in protein content was greater in GG25 (19.37%) and lowest in GG22 (4.68%). The increase in protein content of the germinated grains is augmented by the loss of chemical components. Carbohydrates, proteins and fats are utilized during respiration and increase the availability of amino acids during germination^35,36^.

The crude fat content of the non-germinated green gram cultures ranged between 1.79 and 2.79 g 100 g^-1,^ which varied significantly (*p<0.05*) among the optimized sprouted green gram cultures in the range of 0.52 to 1.4 g 100 g^-1,^ and the percentage loss of change ranged from 47.79% (GG25) to 77.29% (GG22) compared with that of the raw green gram cultures. These results were consistent with those of the study of El-Adawy *et al.*^38^^,^ who reported that the fat content decreased from 1.75 to 1.28 g 100 g^-1^ in mung beans, from 2.40 to 1.77 g 100 g^-1^ in peas and from 1.15 to 0.93 g 100 g^-1^ in lentils after germination. The fat content is reduced due to hydrolysis and the utilization of fat for energy sources and biochemical processes at the time of germination^39,40^.

The high fiber content of green grams makes them a healthy option contributing to improved gut health and other functional health benefits. The crude fiber content of non-germinated green grams ranged from 9.86 to 13.82 g 100 g^-1^ sample, whereas in 33 reports, the fiber content was 4.12 and 4.07% in the GC16 and GC20 green gram lines, respectively. Similarly, compared with those of raw non-germinated green cultures, the fiber content of sprouted green gram cultures varied significantly from 13.86 to 16.42 g 100 g^-1,^ and the percentage increase in fiber content varied between 2.82% and 46.06%. The present findings align with those reported by Megat *et al.*^41^, who observed notable increases in total dietary fiber during germination: from 37% to 60% in kidney beans, 29% to 32% in mung beans, 32% to 73% in soybean, and 27% to 40% in peanuts. Previous studies^42,43^ suggested that the loss of dry matter due to enzymatic starch hydrolysis and the degradation of cellular constituents, including proteins, fats, and carbohydrates, could account for up to 456% of the fiber increase in lupin and the entire 100% increase in peas. Additionally, the rise in fiber content may be attributed to structural modifications in plant cells during germination. Crude fiber components - such as cellulose, lignin, and hemicelluloses - tend to accumulate significantly during sprouting as the developing plant synthesizes new cellular materials required for shoot growth^40,44^.

The ash content of the pulses is indicated since pulses provide a good amount of minerals. The ash content of the non-germinated and germinated green gram cultures ranged between 1.56 and 2.42 g 100 g^-1^ and between 1.61 and 2.50 g 100 g^-1,^ respectively. This finding is in agreement with the results of previous studies of green gram bacteria reported by Agugo *et al.*^33^ Sprouted green gram cultures had ash contents that increased from 0.49% in GG22 to 4.28% in GG29 compared with those of non-germinated green grams. Similar results were observed in studies of the germination of green grams, lentils, and peas^38^. The increase in ash content may be due to the breakdown of anti-nutrient compounds due to phytase activity during the germination process, which reduces phytic acid levels, which bind minerals and render them unavailable for absorption^30^.

Green gram cultures have negligible vitamin C content, but a phenomenal linear increase in vitamin C levels was observed during the germination process. These results are in agreement with those of Masood *et al.*^45^. The vitamin C content significantly varied from 55.04 (GG29) to 85.58 mg 100 g^-1^ (GG25) among the sprouted green gram samples. A high vitamin C content was observed in GG25 (85.58 mg 100 g^-1^), which was on par with that in GG22 (85.48 mg 100 g^-1^) and significantly greater than that in GG28 (56.83 mg 100 g^-1^) and GG29 (55.04 mg 100 g^-1^). The vitamin C content of the sprouted green gram was consistent with the results of previous studies of finger millet, wheat, green gram and chickpea^46,47^. An elevation in vitamin C levels during germination is facilitated by enzymatic starch degradation through the activity of amylases and diastases. This process increases glucose availability, which in turn acts as a key substrate for the biosynthetic pathway of vitamin C^46^. However, the differences in the nutrient characteristics of the selected green gram cultivars varied according to the cultivation location, climatic conditions, cultivation practices, and variety of green gram. Similarly, our results for the physicochemical components among the selected green gram cultures were associated with the physicochemical components of the Indian cultivars of green gram varieties^3,48^ and the South Korean green gram varieties^49^.

### Anti-nutritional factors present in the selected green gram culture

The changes in the levels of anti-nutritional compounds such as tannins and phytic acid in the non-germinated and optimized germinated samples of the selected green gram cultures were assessed, and the results are shown in Table 2 and Table 3, respectively. The tannin content of the selected non-germinated green gram cultures ranged from 320 to 458 TAE mg^-1^. On germination of the selected green-light cultures, the tannin content decreased and ranged from 65 to 97 TAE mg^-1^. The tannin content was significantly lower in the germinated green gram cultures than in the respective non-germinated green gram cultures, which may be due to the soaking treatment and germination stage. The phytic acid content of the raw green gram culture was estimated to range between 906 and 754 mg 100 g^-1,^ whereas the phytic acid content in the respective germinated green gram cultures ranged from 102 to 175 mg 100 g^-1^. Tannin and phytates are water-soluble phenol compounds that are primarily present in the seed coats of legumes. During the germination process, the anti-nutrition compounds are leached from the green gram by stimulating enzymatic activity. The obtained results were in agreement with the results of^45,50^.

### Phytochemical composition of the selected green‒green culture materials

Green grams contain numerous phytochemical compounds and phenolic compounds, which are the main contributors to antioxidant activity and have also been shown to promote health^51^. The phytochemical characteristics of the non-germinated and germinated green gram cultures are presented in Table 4 and Table 5, respectively.

**Table 4:** Phytochemical composition of non-germinated green gram cultures

**Green gram culture**	**Phytochemical characteristics (100g**^**-1**^**)**		
	**Total antioxidant activity (% RSA)**	**Total phenols** **(GAE mg)**	**Total flavonoids** **(QAE mg)**
GG21	26.31±0.34^d^	67.3±2.18^b^	21.4±0.04^g^
GG22	24.24±0.93^e^	48.6±0.26^de^	22.2±0.21^efg^
GG23	28.90±1.09^bc^	49.7±1.61^d^	27.5±0.56^b^
GG24	29.80±0.45^b^	64.2±0.98^c^	20.4±0.77^h^
GG25	23.78±0.55^e^	19.0±0.73^f^	21.8±0.47^fg^
GG26	32.41±1.16^a^	47.8±0.30^e^	24.6±0.35^d^
GG27	29.18±0.76^b^	77.8±0.08^a^	35.4±0.35^a^
GG28	28.52±0.82^bc^	62.6±0.50^c^	22.8±0.63^e^
GG29	26.75±0.96^d^	58.8±0.15^d^	22.6±0.34^ef^
GG30	27.48±0.54^cd^	60.0±0.54^d^	25.8±0.62^c^

GAE – Gallic acid equivalent; QAE – Quercetin acid equivalent; % RAS – Percentage of Radical Scavenging Activity; Each result is expressed as the mean ± standard deviation (SD) of three independent replicates (n = 3). Superscript letters indicate values within the same column that differ significantly at *p* < 0.05.

**Table 5:** Phytochemical composition of germinated green gram cultures

**Green gram culture**	**Phytochemical characteristics (100g)**		
	**Total antioxidant activity** **(% RSA)**	**Total phenols** **(GAE mg)**	**Total flavonoids** **(QAE mg)**
GG21	30.81±0.57^e^	74.2±1.51^b^	42±1.15^e^
GG22	36.00±0.94^d^	53.8±2.07^f^	45±0.60^cd^
GG23	38.50±1.14^b^	64±0.69^d^	51.5±0.51^b^
GG24	38.94±0.17^b^	72.6±0.09^bc^	33.8±1.30^g^
GG25	35.47±1.08^d^	32.4±1.11^g^	52.5±0.47^b^
GG26	40.59±0.62^a^	58.4±1.07^ef^	43.5±1.72^de^
GG27	37.98±0.75^bc^	80.4±1.90^a^	57.3±0.08^a^
GG28	37.73±1.29^bc^	68.8±1.53^c^	46.5±1.34^c^
GG29	36.56±0.09^c^	68.2±0.59^c^	38.5±1.49^f^
GG30	35.91±0.29d	64.5±0.16^d^	41.8±1.65^e^

# Fresh sprouted green gram samples; GAE – Gallic acid equivalent; QAE – Quercetin acid equivalent; % RSA – Percentage of Radical Scavenging Activity; Each result is expressed as the mean ± standard deviation (SD) of three independent replicates (n = 3). Superscript letters indicate values within the same column that differ significantly at *p* < 0.05.

Total phenolic compound levels, in terms of FAE and GAE, were significantly different between the green gram cultures and ranged from 35-140 mg of FAE 100 g^-1^ to 19.0-77.8 mg of GAE 100 g^-1,^ with the highest value occurring in GG27 and the lowest occurring in GG25. After germination, the total phenolic content ranged from 72.4-163.5 mg of FAE to 32.4-80.4 mg of GAE 100 g^-1^. The FAEs and GAEs of the total phenolics were significantly different between the cultures of the sprouted green grams. Significant increases were observed in GG25 (106.86% of FAEs and 70.53% of GAEs), and the lowest changes were observed in GG24 (6% of FAEs and 13.08% of GAEs) and GG27 (16.79% of FAEs and 3.34% of GAEs).

The total flavonoid content in the non-germinated green gram cultures ranged significantly (*p* < 0.05) between 20.4–35.4 QAE mg of 100 g^-1^ and 6.6–34.6 CAE mg of 100 g^-1^. In comparison, the sprouted cultures showed markedly higher values, ranging from 33.8–57.3 QAE mg of 100 g^-1^ to 19.5–51.5 CAE mg of 100 g^-1^. Thus, germination led to a significant enhancement in the flavonoid content of green gram.

The total antioxidant activity of the non-germinated green gram cultures was significantly different, and the percentage of RSA ranged between 23.78% in GG25 and 32.41% in GG26. An increase in total phenols, total flavonoids and vitamin C may influence the antioxidant properties of sprouted green grams. The total antioxidant activity of the sprouted green gram was found to increase significantly among the non-germinated green gram cultures, and the percentage of inhibition ranged between 35.47% in GG25, which was on par with that in GG22 (36%), and that in GG30 (35.91%), which was 40.59%. The percentage of inhibition increased in the range of 17.10 to 49.16% in the germinated green gram cultures compared with the raw green gram cultures. Similar results were observed for total flavonoid content, phenolic content and antioxidant activity in germinated mung beans^50,52,53^. Phenylalanine ammonia-lyase activity is enhanced during the germination process and is responsible for increasing the rate of biosynthesis of phenols and flavonoids; another explanation may be that enzymes hydrolyze bound phenols as well as biosynthesize phenols in sprouts^54,55^.

### Functional properties of selected green gram cultures

**Fig 3:**
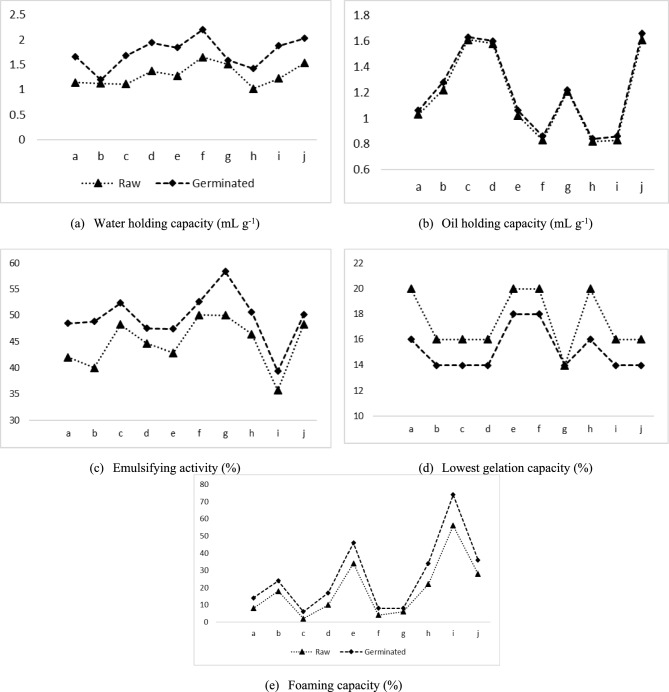
Functional characteristics of the non-germinated and germinated green gram cultures **a**(GG21), **b**(GG22), **c**(GG23), **d**(GG24), **e**(GG25), **f**(GG26), **g**(GG27), **h**(GG28), **i**(GG29) and j(GG30); Water holding capacity, oil holding capacity, emulsifying activity, lowest gelation capacity and foaming capacity values are the mean values of three replicate analysis (n=3)

The results of the functional property analyses, such as WHC, OHC, LGC, EA and GC, of the green gram cultures are presented in Fig. 3. The WHC and OHC varied significantly (*p<0.05*) between the flour samples of the green gram cultures. The flours exhibited WHCs and OHCs of 1.02-1.65 mL g^-1^ and 0.83 mL g^-1^ to 1.61 mL g^-1^, respectively, wherein a significantly greater WHC was obtained for GG26 and OAC was obtained for GG30. Additionally, the WHC and OHC were significantly (*p<0.05*) greater in the sprouted green gram samples than in the non-germinated samples. The WHC varied from 1.20 to 2.20 mL g^-1^ in the sprouted green gram flour. Similarly, the OHC increased among the sprouted samples, with values ranging from 0.84 to 1.66 mL g^-1^. The observed changes in the OHC density increased from 0.83% in GG27 to 4.91 in GG22 in the non-germinated green gram cultures. These values are consistent with the results for the Indian cultivar of green gram varieties. A higher WHC reflects its impact on the functional and sensory properties of food products and is associated with the abundance of hydrophilic residues present in the carbohydrate and protein fractions of pulse flours^3^. Oil absorption, on the other hand, occurs through capillary action and physical entrapment within the flour matrix. Variations in OAC can be attributed to differences in particle size, protein type and proportion, the presence of nonpolar amino acid side chains, and starch composition^29,56^.

The GC content varied between 2-56% and 6-74% in the non-germinated and germinated green gram cultures, respectively. A greater amount of GC was detected in GG29 than in the GG23 culture of raw green gram flour. The optimized germinated green gram culture FC values were significantly greater (33.33%) in GG22 to 200% in GG23 than in the non-germinated green gram cultures. The variation in FC can be explained by the higher ability of certain proteins to diffuse quickly to the air–water interface, thereby improving foam formation^40^. However, the values observed in this study were notably lower than those reported by Wani et al.^3^ for other green gram strains. Enhancement of foaming properties is largely influenced by the solubility of proteins in the flour, which facilitates a reduction in surface tension at the air–water boundary. Another explanation was that the foaming capacity increases with increasing protein solubility, which in turn increases the number of positively charged amino groups during germination^38,40^.

The EC of the raw green gram cultures ranged from 35.71 (GG29) to 50.1% (GG26). The sprouted green gram flour produced a 3.87 to 21.95% (39.41% to 58.42%) increase in emulsification capacity compared with that of the non-germinated green gram cultures. The emulsifying capacity increased during the germination of mung beans. The increase in emulsifying capacity was caused by the conversion of oligomeric proteins to simple proteins, and the synthesis of new proteins during germination may increase the amount of soluble proteins, which are more surface active and promote emulsification as oil in water.

The raw and germinated green gram cultures did not show good gelation capacity since they exhibited values of 14, 16, and 20 in the non-germinated samples and 14, 16, and 18 in the sprouted samples. These values are consistent with those of other studies in raw cowpea, jack bean and Mucina^57^. Changes in the chemical composition of the proteins, carbohydrates, and lipids in the flours may influence the formation of a gel.

## Conclusion

Optimized germination represents a simple and effective approach to improving the nutritional quality, functional properties, and antioxidant potential of green gram. The enhancement of bioactive compounds alongside reduced anti nutritional factors highlights the role of germination in improving nutrient bioavailability. These improvements, combined with good consumer acceptability in selected culture and optimized germination, support the use of green gram sprouts as salads in weaning/supplementary foods, nutrient-dense convenience food mixes, formulations of functional foods, etc., for improved nutritional value.

## Data Availability

The authors confirm that the data supporting the findings of this study are available within the article.
